# Age-Specific Risk Scores Do Not Improve HIV-1 Prediction Among Women in South Africa

**DOI:** 10.1097/QAI.0000000000002436

**Published:** 2020-07-23

**Authors:** Kathryn Peebles, Thesla Palanee-Phillips, Jennifer E. Balkus, Ivana Beesham, Heeran Makkan, Jennifer Deese, Jennifer Smit, Renee Heffron, Charles S. Morrison, Neena M. Philip, Mookho Malahleha, Margaret Kasaro, Yuthika Naidoo, Tanya Nielson, Krishnaveni Reddy, Philip Kotze, Khatija Ahmed, Helen Rees, Jared M. Baeten, Ruanne V. Barnabas

**Affiliations:** a Department of Epidemiology, University of Washington, Seattle, WA;; b Wits Reproductive Health and HIV Institute, School of Clinical Medicine, University of the Witwatersrand, Johannesburg, South Africa;; c Department of Global Health, University of Washington, Seattle, WA;; d Vaccine and Infectious Disease Division, Fred Hutchinson Cancer Research Center, Seattle, WA;; e MatCH Research Unit (MRU), Department of Obstetrics and Gynaecology, Faculty of Health Sciences, University of the Witwatersrand, Durban, South Africa;; f Clinical Research Division, The Aurum Institute, Johannesburg, South Africa;; g Advancing Care and Treatment for TB/HIV, A Collaborating Centre of the South African Medical Research Council, South Africa;; h Global Health, Population and Nutrition, FHI 360, Durham, NC;; i ICAP at Columbia University, Mailman School of Public Health, New York, NY;; j Setshaba Research Centre, Tshwane City, South Africa;; k University of North Carolina, Global Projects Zambia, Lusaka, Zambia;; l Qhakaza Mbokodo Research Clinic, Ladysmith, South Africa; and; m Department of Medicine, University of Washington, Seattle, WA.

**Keywords:** sub-Saharan Africa, South Africa, empiric risk scoring tool, HIV-1, women

## Abstract

HIV-1 risk scoring tools could help target provision of prevention modalities such as pre-exposure prophylaxis. Recent research suggests that risk scores for women aged 18–45 may not predict risk well among young women aged 18–24. We evaluated the predictive performance of age-specific risk scores compared with the existing non–age-specific VOICE risk score, developed for women aged 18–45.

**Methods:**

We conducted a secondary analysis of the Evidence for Contraceptive Options and HIV Outcomes Trial to develop and internally validate HIV-1 risk scores for women aged 18–24 and 25–35 in South Africa. Candidate predictors included baseline demographic, clinical, behavioral, and contextual characteristics readily available in clinical settings. The VOICE risk score was applied to women aged 18–35. We evaluated predictive performance of each risk score by area under the receiver operating characteristic curve (AUC).

**Results:**

Predictive performance of all risk scores was moderate, with AUC (95% confidence interval) of 0.64 (0.60 to 0.67) among women aged 18–24, 0.68 (0.62 to 0.73) among those aged 25–35, and 0.61 (0.58 to 0.65) for the VOICE risk score applied to women aged 18–35; The AUC was similar in internal validation. Among women aged 18–24, HIV-1 incidence was high even at low risk scores, at 3.9 per 100 person-years (95% confidence interval: 3.2 to 4.7).

**Conclusions:**

All risk scores were moderately predictive of HIV-1 acquisition, and age-specific risk scores performed only marginally better than the VOICE non–age-specific risk score. Approaches for targeted pre-exposure prophylaxis provision to women in South Africa may require more extensive data than are currently available to improve prediction.

## Abstract 2

Supplemental Digital Content is Available in the Text.

## INTRODUCTION

Women account for more than half of new HIV-1 infections in sub-Saharan Africa, with an even more marked disparity in risk among adolescent girls and young women, who account for 70% of new infections among individuals aged 15–24.^1^ Pre-exposure prophylaxis (PrEP) is a highly effective HIV-1 prevention method, reducing risk by more than 90% when used with high adherence.^2^ With increasing availability in sub-Saharan Africa, PrEP has the potential to contribute significantly to HIV-1 prevention goals in the region.^3^ However, strategies to efficiently promote and allocate PrEP to those at highest risk are needed to ensure maximal impact within resource constraints.

Empirically derived risk scoring tools are one potential strategy to help identify those who might benefit most from PrEP. Risk scoring tools to identify those at greatest risk for HIV-1 have been developed for a number of settings and populations, including for men who have sex with men in the United States^4–6^ and for serodiscordant couples,^7^ pregnant and postpartum women,^8^ and women aged 18–45 in sub-Saharan Africa.^9^ In previous research, these risk scores have been associated with self-perceived HIV-1 risk,^10^ which is in turn associated with higher PrEP uptake.^11,12^ Nonetheless, in both the United States and sub-Saharan Africa, only approximately one-third of those identified to be at high risk of HIV-1 acquisition by risk score evaluation had self-perceived high risk.^13,14^ Qualitative research and pilot studies suggest that high risk scores may provide an opportunity to reassess self-perceived low risk and prompt engagement in protective behaviors.^15,16^ Use of risk scores to inform prioritization of PrEP provision could thus serve to increase both efficiency and overall uptake of PrEP.

Risk scores are population‐specific and setting‐specific, often performing well only among groups comparable to the population in which the risk score was initially developed.^17,18^ The MTN-003/VOICE risk score was previously developed and externally validated in 3 cohorts of women aged 18–45 participating in clinical trials of HIV-1 prevention products in sub-Saharan Africa.^9,19^ However, the VOICE risk score performed poorly among adolescent girls and young women in recent research,^20^ suggesting that a different set of risk factors may be more predictive of HIV-1 acquisition among younger age groups. Given high HIV-1 incidence in this group of women,^1^ risk scores tailored to their unique risk profile are needed.

We conducted a secondary analysis of the Evidence for Contraceptive Options and HIV Outcomes (ECHO) Trial.^21^ This study enrolled 3603 young women aged 18–24 and 2165 women aged 25–35 in diverse geographic settings in South Africa, providing a unique opportunity to (1) develop and evaluate the predictive performance of age-specific risk scores, compared with the predictive performance of the non–age-specific VOICE risk score and (2) investigate whether the components of age-specific risk scores differed among women aged 18–24 and women aged 25–35.

## METHODS

The ECHO Trial was a randomized trial of 7829 HIV–1-negative women seeking effective contraception in Eswatini, Kenya, South Africa, and Zambia from 2015 to 2018; detailed methods and results have been published previously.^21^ As most study participants were from South Africa, we limited development of the risk score to women enrolled in the 9 sites in South Africa, representing a geographically diverse range of settings across 5 provinces. The primary endpoint of analyses was incident HIV-1 infection; we therefore limited the analysis to women confirmed HIV‐1‐negative at enrollment and with at least 1 follow-up HIV-1 test. Institutional review boards at each site approved the study protocol, and women provided written informed consent.

We used Cochran–Mantel–Haenszel χ^2^ tests stratified by study site to evaluate differences in the distribution of participant characteristics among women aged 18–24 and 25–35. We used standard methods^22^ to develop and internally validate risk scores for women aged 18–24 and 25–35. Follow-up time was censored at 1 year (within a window of 2 weeks before the scheduled annual visit and up to 11 weeks after) because shorter-term outcomes are most relevant for decision-making regarding short-acting prevention methods, such as PrEP. We aimed to develop risk scores that could be applied in clinical settings in sub-Saharan Africa and therefore considered as candidate predictors 25 baseline demographic, clinical, behavioral, and contextual (defined as HIV-1 prevalence of the surrounding area or province) characteristics that could be readily available in clinical settings. Given that laboratory evaluation of sexually transmitted infections (STI) in these settings is not common, we additionally developed modified risk scores excluding laboratory-based variables. We used a categorical parameterization for continuous variables if the predictive performance with such parameterization, defined by area under the receiver operating characteristic curve (AUC), was comparable with the continuous parameterization or if the continuous parameterization would be infeasible in practice (eg, continuous number of partners may result in a risk score with too many points to be easily computed). Categorization of continuous variables was determined by the dichotomous cutpoint that maximized Youden's J statistic.^23^ We estimated site-specific HIV-1 prevalence in 5% increments from 10% to 30% from publicly available prevalence microdata.^24^ We evaluated the association between each candidate predictor and incident HIV-1 with Cox proportional hazards models, excluding women with incomplete baseline data. Variables associated with incident HIV-1 with statistical significance *P* < 0.10 were included in a fully stepwise multivariable Cox proportional hazards model. We selected the model with the lowest Akaike information criterion^25^ as the final risk score model and assigned points to individual variables by dividing each coefficient by the smallest coefficient among all variables in the model and rounding to the nearest integer. Although our goal was to create a tool that could be easily used in clinical settings, information may be lost both in the categorization of continuous variables and in calculating rounded point values. We therefore evaluated the potential loss of predictive performance when using a simplified tool by additionally characterizing the AUC of the full multivariable model with continuously parameterized variables.

We evaluated model calibration by graphically comparing observed and risk score–predicted cumulative HIV-1 incidence by risk score value.^22^ We calculated the AUC to evaluate overall predictive performance of the risk score^26^ and identified the optimal risk score threshold value as the value that maximized Youden's J statistic. If HIV-1 incidence among those not meeting the optimal threshold value exceeded the World Health Organization (WHO)-recommended threshold for PrEP use of 3 per 100 person-years,^27^ we also identified an alternative threshold below which incidence was less than 3 per 100 person-years. We additionally compared the predictive performance of the full risk score with the predictive performance of each individual predictor. For each risk score, we calculated the sensitivity, specificity, and positive and negative predictive values. We performed internal validation by repeating the full risk score development process in 100 bootstrapped data sets; use of this approach minimizes the risk of model over-fitting.^22^

We performed external validation of the VOICE risk score^9^ by evaluating its predictive performance among women aged 18–35 in South Africa. The VOICE risk score was developed from a cohort of women in South Africa, Uganda, and Zimbabwe and includes as predictors age, marital and cohabitation status, alcohol use in the previous 3 months, receipt of financial or material support from a partner, whether a partner has other sex partners, any curable STI, and herpes simplex virus type 2 (HSV-2).^9^ Although the VOICE risk score includes alcohol consumption in the previous 3 months, alcohol use in ECHO was collected as the number of weekly drinks. We therefore used an indicator of any weekly alcohol consumption in place of alcohol consumption in the previous 3 months in applications of the VOICE risk score to these cohorts. All analyses were conducted in R^28^ version 3.5.0.

## RESULTS

### Study Population

Analyses include 5573 women aged 18–35 from South Africa. Women aged 25–35 were more likely than those aged 18–24 to be married or living with a partner, earning an income, and receiving financial and/or material support from a partner (all *P* < 0.001; Table 1). Infection with *Chlamydia trachomatis* at enrollment was more common among women aged 18–24, whereas women aged 25–35 were nearly 2‐fold more likely to test positive for HSV-2 (both *P* < 0.001; Table 1). The median follow-up time with censoring at the twelve-month visit was 364 days (interquartile range: 364–368).

**TABLE 1.** Baseline Characteristics Among Women Enrolled in ECHO in South Africa, n = 5573*

Characteristic	Ages 18–24, n = 3461	Ages 25–35, n = 2112	*P*†
Married or living with partner	294 (8.5)	607 (28.7)	<0.001
Educational attainment			
None or any primary	6 (0.2)	17 (0.8)	<0.001
Any secondary	2825 (81.6)	1777 (84.1)	
Postsecondary	630 (18.2)	318 (15.1)	
Earns own income	488 (14.1)	587 (27.8)	<0.001
Receives material and/or financial support from partner	1671 (48.3)	1324 (62.7)	<0.001
Any weekly alcohol consumption	745 (21.5)	475 (22.5)	0.319
More than one sex partner in the previous 3 mo	284 (8.2)	178 (8.4)	0.981
Partner has sex with others			
No	1119 (32.3)	617 (29.4)	<0.001
Do not know	618 (17.9)	344 (16.4)	
Yes	1724 (49.8)	1141 (54.3)	
Condom use frequency			
Never or rarely	807 (23.3)	643 (30.6)	<0.001
Sometimes, often, or always	2654 (76.7)	1458 (69.4)	
*Neisseria gonorrhoeae*	202 (5.8)	88 (4.2)	0.017
*Chlamydia trachomatis*	888 (25.7)	303 (14.3)	<0.001
HSV-2 status‡	1363 (39.4)	1412 (66.9)	<0.001

All Values are n (%).

* Among 5670 enrolled in South Africa, 97 (1.7%) are excluded from analyses because of missingness in variables included in multivariable prediction models.

† Obtained from Cochran–Mantel–Haenszel χ^2^ test stratified by the study site.

‡ Defined as a HSV-2 enzyme immunoassay index value of greater than or equal to 0.90.

HSV-2, herpes simplex virus type 2.

### Risk Scores Among Women Aged 18–24

**FIGURE 1.**
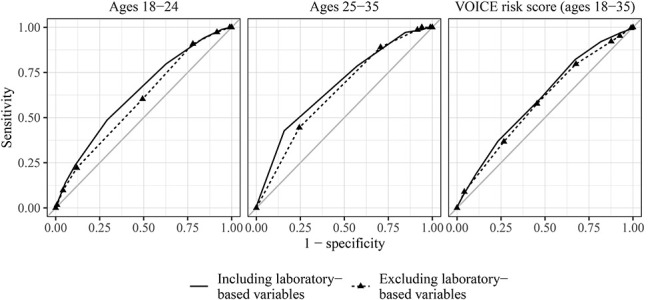
Receiver operating characteristic curves for age-specific and VOICE risk scores including and excluding laboratory-based variables. The gray line indicates a risk score with the area under the curve of 0.50, indicating no predictive ability.

In the first year of ECHO follow-up in South Africa, 188 women aged 18–24 acquired HIV-1, at an incidence rate of 5.4 per 100 person-years [95% confidence interval (CI): 4.6 to 6.2]. Factors associated with HIV-1 at the significance level *P* < 0.10 in univariate analyses were reported condom use frequency, marital and cohabitation status, number of sex partners in the previous 3 months, whether a primary partner has other sex partners, alcohol consumption, HIV-1 prevalence, *Neisseria gonorrhoeae*, *C. trachomatis*, and HSV-2. Of these, all but marital and cohabitation status and *C. trachomatis* were retained in the final stepwise model and risk score (Table 2). *N. gonorrhoeae* was weighted most heavily in the final risk score, with 3 points, followed by receiving services in an area with HIV-1 prevalence greater than 15% and having more than one sex partner in the previous 3 months (2 points each). Nonetheless, the full risk score performed better than each of the individual predictors (see Figure S1, Supplemental Digital Content 1, http://links.lww.com/QAI/B502). The AUC for the full risk score was 0.64 (95% CI: 0.60 to 0.67), indicating modest predictive ability (Fig. 1). In internal validation, the AUC was 0.62 (95% CI: 0.58 to 0.64) across 100 bootstrapped resamples in which the full model selection procedure was repeated.

**TABLE 2.** Association Between Select Baseline Predictors and HIV-1 Incidence From Multivariable and Stepwise Models and Resulting Risk Score Points

Characteristic*	Ages 18–24					Ages 25–35				
	Hazard Ratio (95% CI)†	Coefficient‡	Risk Score points‡	Coefficient§	Risk Score points§	Hazard Ratio (95% CI)†	Coefficient‡	Risk Score points‡	Coefficient§	Risk Score points§
Age										
Less than 27	—	—	—	—	—	2.12 (1.31 to 3.41)	0.776	1	0.668	1
27 and above	—	—	—	—	—	Reference	—	—	—	—
Marital/cohabitation status										
Married or living with partner	Reference	—	—	—	—	Reference	—	—	—	—
Not married nor living with partner	1.57 (0.80 to 3.09)	—	—	—	—	1.85 (1.07 to 3.31)	0.64	1	0.728	1
Weekly alcohol consumption										
None	Reference	—	—	—	—	—	—	—	—	—
One or more drinks	1.45 (1.03 to 2.05)	0.375	1	0.430	1	—	—	—	—	—
HIV-1 prevalence										
10%–15%	Reference	—	—	—	—	—	—	—	—	—
16%–20%	1.64 (1.08 to 2.48)	0.485	2	0.449	2	—	—	—	—	—
21%–25%	1.71 (0.99 to 2.96)	0.565	2	0.538	2	—	—	—	—	—
26%–30%	1.81 (1.03 to 3.19)	0.602	2	0.610	2	—	—	—	—	—
Province										
Western Cape	—	—	—	—	—	Reference	—	—	—	—
Eastern Cape	—	—	—	—	—	9.05 (1.18 to 69.2)	2.203	3	2.169	3
KwaZulu-Natal	—	—	—	—	—	6.37 (0.87 to 46.84)	1.792	3	1.791	3
Gauteng	—	—	—	—	—	5.81 (0.78 to 43.31)	2.047	3	1.995	3
North West	—	—	—	—	—	7.94 (0.99 to 63.56)	2.163	3	2.159	3
No. of sex partners in previous 3 mo										
None or one	Reference	—	—	—	—	—	—	—	—	—
More than one	1.61 (1.06 to 2.44)	0.491	2	0.565	2	—	—	—	—	—
Partner has sex with others										
No	Reference	—	—	—	—	—	—	—	—	—
Yes or do not know	1.31 (0.93 to 1.85)	0.291	1	0.346	1	—	—	—	—	—
Condom use										
Never or rarely	Reference	—	—	—	—	—	—	—	—	—
Sometimes, often, or always	1.34 (0.92 to 1.95)	0.316	1	0.291	1	—	—	—	—	—
*N. gonorrhoeae*	2.07 (1.33 to 3.24)	0.788	3	—	—	2.22 (0.99 to 5.00)	0.913	1	—	—
*C. Trachomatis*	1.22 (0.89 to 1.67)	—	—	—	—	1.42 (0.79 to 2.54)	—	—	—	—
HSV-2 positive║	1.51 (1.13 to 2.02)	0.411	1	—	—	1.88 (1.07 to 3.31)	0.631	1	—	—

* Other factors evaluated include number of previous pregnancies (continuous and categorical), number of living children (continuous and categorical), desire for future children, vaginal sex in the past week, vaginal sex in the past 2 weeks, number of vaginal sex acts in the past week, vaginal sex during menses in the previous 3 months, anal sex in the previous 3 months, partner circumcision status, partner HIV-1 status, educational attainment, and presence of vaginal discharge.

† Hazard ratio from a multivariable Cox proportional hazards model.

‡ Coefficients and risk score points from the final model of a fully stepwise model selection procedure.

§ Coefficients and risk score points from a modified fully stepwise model excluding laboratory-based variables.

║ Defined as a HSV-2 enzyme immunoassay index value of greater than or equal to 0.90.

HSV-2, herpes simplex virus type 2.

**FIGURE 2.**
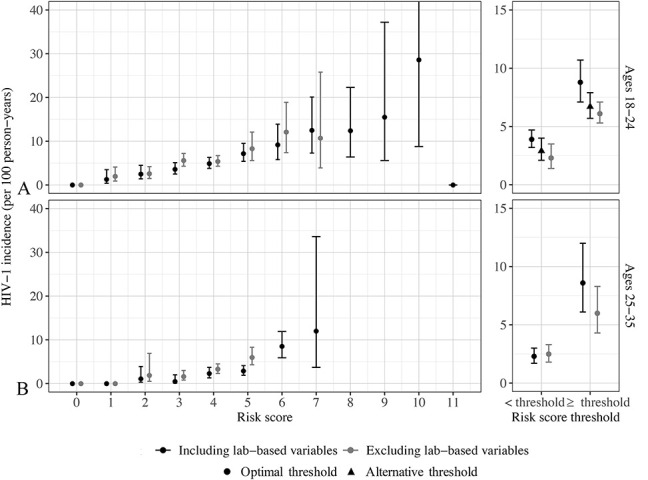
HIV-1 incidence by risk score points and threshold values in risk scores including or excluding laboratory-based STI variables among (A) women aged 18–24 and (B) women aged 25–35. Among women aged 18–24 with 10 points on the full risk score, the CI extends to 69.6 cases per 100 person-years; y axes are restricted to a maximum incidence of 40 cases per 100 person-years to allow interpretability of all plotted points. The optimal risk score threshold for women aged 18–24, including lab-based variables, is 5 points, with an alternative threshold of 4 points. In a risk score excluding lab-based variables for women aged 18–24, the optimal threshold is 4. Among women aged 25–35, the optimal threshold is 6 for the full risk score and 5 for a modified risk score excluding lab-based variables.

Across all risk scores, there was a dose–response relationship in HIV-1 incidence with increasing risk score points (Fig. 2A; see Table S1, Supplemental Digital Content 1, http://links.lww.com/QAI/B502). There was a similar dose–response relationship in risk score-predicted HIV-1 incidence, indicating good calibration of risk scores (see Figure S2, Supplemental Digital Content 1, http://links.lww.com/QAI/B502). Among women aged 18–24, incidence ranged from zero cases among women with zero points in the full risk score including laboratory-based variables to 25.0 (95% CI: 7.7 to 69.6) per 100 person-years among women with 10 or more points. Women meeting the optimal threshold value of ≥5 points experienced HIV-1 incidence of 8.8 (95% CI: 7.1 to 10.7) per 100 person-years, whereas incidence among women with <5 points was approximately halved at 3.9 (95% CI: 3.2 to 4.7) per 100 person-years (see Figure S3, Supplemental Digital Content 1, http://links.lww.com/QAI/B502). At an alternative threshold of ≥4 points, HIV-1 incidence was 6.7 (95% CI: 5.7 to 7.9) among those screening positive and 2.9 (95% CI: 2.1 to 4.0) per 100 person-years among those with a risk score of fewer than 4 points. Thirty percent of women aged 18–24 met the optimal threshold value of ≥5 points, accounting for 48.6% of infections among all women aged 18–24 (Table 3). Sensitivity was higher at the alternative threshold of ≥4 points, with a concomitant decline in specificity (Table 3).

**TABLE 3.** Predictive Performance Characteristics of Age-Specific Risk Scores Including and Excluding Laboratory-Based Variables

Risk Score Points	Sensitivity	Specificity	Positive Predictive Value	Negative Predictive Value	Sensitivity	Specificity	Positive Predictive Value	Negative Predictive Value
	Including laboratory-based variables				Excluding laboratory-based variables			
Ages 18–24								
1	1.000	0.006	0.054	1.000	1.000	0.011	0.054	1.000
2	0.989	0.054	0.056	0.989	0.973	0.084	0.056	0.982
3	0.941	0.159	0.060	0.979	0.908	0.220	0.062	0.977
4	0.798	0.373	0.067	0.970	0.603	0.506	0.064	0.958
5	0.486	0.708	0.086	0.960	0.221	0.880	0.094	0.953
6	0.236	0.890	0.108	0.954	0.098	0.958	0.117	0.950
7	0.138	0.946	0.126	0.951	0.017	0.992	0.110	0.947
8	0.067	0.974	0.130	0.948	—	—	—	—
9	0.024	0.992	0.151	0.947	—	—	—	—
10	0.007	0.998	0.153	0.947	—	—	—	—
11	0.000	1.000	0.000	0.946	—	—	—	—
Ages 25–35								
1	1.000	0.002	0.034	1.000	1.000	0.011	0.035	1.000
2	1.000	0.017	0.034	1.000	1.000	0.060	0.036	1.000
3	0.986	0.063	0.035	0.992	0.986	0.085	0.037	0.994
4	0.972	0.153	0.038	0.994	0.890	0.294	0.043	0.987
5	0.786	0.427	0.046	0.983	0.445	0.756	0.061	0.975
6	0.427	0.842	0.086	0.977	—	—	—	—
7	0.028	0.993	0.118	0.967	—	—	—	—

A modified risk score for women aged 18–24 excluding laboratory-based variables was similar to the full risk score (Table 2), but had somewhat poorer predictive performance, with an AUC of 0.60 (95% CI: 0.57 to 0.62) (Fig. 1). In internal validation, the AUC across 100 bootstrapped resamples was 0.59 (95% CI: 0.55 to 0.61). HIV-1 incidence ranged from zero cases among women with zero risk score points to 11.8 (95% CI: 7.5 to 17.9) per 100 person-years among those with 6 or more risk score points (Fig. 2B; see Table S1, Supplemental Digital Content 1, http://links.lww.com/QAI/B502). At the optimal threshold value of ≥3 points, incidence was 6.1 (95% CI: 5.3 to 7.1) per 100 person-years, compared with 2.3 (95% CI: 1.4 to 3.5) among women with fewer than 3 risk score points (see Figure S3, Supplemental Digital Content 1, http://links.lww.com/QAI/B502). Approximately three-quarters of all women aged 18–24 met the optimal threshold value, accounting for 90.8% of incident infections (Table 3). For all risk scores, the AUC was similar between each risk score and its corresponding full multivariable model with continuously parameterized variables (see Figure S4, Supplemental Digital Content 1, http://links.lww.com/QAI/B502).

### Risk Scores Among Women Aged 25–35

HIV-1 incidence in the first year of follow-up among women aged 25–35 was 3.4 per 100 person-years (95% CI: 2.7 to 4.0). A combination of demographic (age and marital and cohabitation status), contextual (province), and clinical (*N. gonorrhoeae*, *C. trachomatis*, and HSV-2) factors were associated with HIV-1 incidence at significance *P* < 0.10 (Table 2). With the exception of *C. trachomatis*, all variables were retained in the final stepwise prediction model. Of these, province was weighted most heavily in risk score points, with 3 points assigned to those living in Eastern Cape, Gauteng, KwaZulu-Natal, and North West provinces, relative to zero points among women in Western province. All other components of the risk score were assigned 1 point each. Similar to the full risk score developed for women aged 18–24, the full risk score for women aged 25–35 performed better than each of its component risk factors (see Figure S1, Supplemental Digital Content 1, http://links.lww.com/QAI/B502). Predictive performance for the full risk score was moderate, with an AUC of 0.68 (95% CI: 0.62 to 0.73) and mean AUC in internal validation of 0.64 (95% CI: 0.59 to 0.69). HIV-1 incidence by risk score points ranged from zero among women with zero risk score points to 12.0 (95% CI: 3.7 to 33.6) per 100 person-years among women with the maximum risk score points of 7. At the optimal threshold value of ≥6 points, incidence was 8.6 per 100 person-years (95% CI: 6.1 to 12.0), approximately 4 fold higher than incidence among women with fewer than 6 risk score points (2.3, 95% CI: 1.7 to 3.0) (see Figure S3, Supplemental Digital Content 1, http://links.lww.com/QAI/B502). Women aged 25–35 with ≥6 risk score points accounted for 42.7% of incident infections (Table 3), yet accounted for only 16.7% of women in this age band.

A modified risk score excluding laboratory-based variables was similar, with all included risk factors retaining the same risk score points as in the full risk score. Predictive performance was slightly lower, with an AUC of 0.64 (95% CI: 0.58 to 0.70) in the full derivation data set and a mean AUC of 0.62 (95% CI: 0.58 to 0.67) across 100 bootstrapped resamples in internal validation. HIV-1 incidence was highest among women with the optimal threshold value of ≥5 points at 6.0 (95% CI: 4.3 to 8.3) per 100 person-years, relative to incidence of 2.5 (95% CI: 1.8 to 3.3) per 100 person-years among women with fewer than 5 risk score points (see Figure S3, Supplemental Digital Content 1, http://links.lww.com/QAI/B502). Approximately one-quarter of women aged 25–35 had 5 risk score points, accounting for 44.5% of incident infections in this age group (Table 3).

### Validation of VOICE Risk Scores Among Women Aged 18–35

The VOICE risk score including laboratory-based variables had moderate predictive performance among South African women aged 18–35, with an AUC of 0.61 (95% CI: 0.58 to 0.65), similar to predictive performance of age-specific risk scores (Fig. 1). Performance of the modified VOICE risk score excluding laboratory variables was slightly lower in South Africa, with an AUC of 0.59 (95% CI: 0.56 to 0.62) (Fig. 1).

## DISCUSSION

We developed and internally validated age-specific HIV-1 risk scores for women in South Africa, identifying both differences and similarities in risk factors by age. Among women aged 18–24, a combination of baseline behavioral, clinical, and contextual factors best predicted subsequent HIV-1 acquisition, whereas among women aged 25–35, demographic, rather than behavioral risk factors combined with clinical and contextual factors to form the optimal risk score. Across all ages, HIV-1 prevalence of the surrounding area was an important predictor of risk, although the full risk score performed better than prevalence alone, emphasizing the importance of both individual and contextual factors in HIV-1 risk. These findings support the multipronged approach to PrEP implementation taken by governments such as Kenya's and South Africa's, where targeting of PrEP provision is guided by both regional prevalence and consideration of key populations, and is further refined in Kenya with use of a rapid screening tool.^29,30^ Such approaches are predicted to both achieve high coverage^31^ and maximize the impact of HIV-1 prevention across regions with heterogeneous HIV-1 prevalence.^32^ In uniformly high-prevalence settings, on the other hand, additional modeling research is needed to evaluate the incremental population-level impact of PrEP provision targeted by individual risk factors.

In both age groups and in the application of the VOICE risk score, the predictive performance of the full risk score was reduced in a modified risk score excluding laboratory-based evaluations of STI. In particular, among young women aged 18–24, *N. gonorrhoeae* was the most heavily weighted risk factor component. In previous research, a history of STIs was associated with PrEP uptake,^13,18^ suggesting that STI diagnoses may be a particularly salient marker of HIV-1 risk for individuals. Inclusion of such objective measures of risk may help potential PrEP users recognize their HIV-1 risk and motivate uptake. However, STI testing in routine clinical care is uncommon in sub-Saharan Africa, posing an important barrier to application of the most useful version of these risk scores and missing opportunities to treat infections associated with increased HIV-1 risk and other morbidities.^33^ Efforts to scale up point-of-care STI tests would increase the feasibility of including such variables in risk scores and thereby optimize their performance and subsequent efficiency of PrEP allocation, particularly for young women.

Age-specific full risk scores developed from the ECHO trial had moderate predictive performance, with the AUC ranging from 0.62 to 0.64 in internal validation. However, HIV-1 incidence among women who did not meet the optimal threshold was still as high as 2.3 per 100 person-years among women aged 25–35, and even higher, at 3.9 per 100 person-years, among women aged 18–24. Elevated HIV-1 incidence among those who would screen negative by risk score emphasizes that these tools are not suitable as eligibility criteria. In the absence of improved predictive performance, a lower risk score threshold, identifying a group of women with incidence exceeding the WHO-recommended threshold for PrEP use of 3 per 100 person-years, is preferable. Although this alternative threshold increased the sensitivity of the risk score to identify women most likely to benefit from PrEP, it also had a higher proportion of false positives, indicating that neither approach is likely to capture the full context of an individual's risk, yet may be important means through which to open dialogue around risk^34^ and set the stage for shared decision-making to select a prevention option.^15^ Integration of PrEP provision with existing services, such as contraception provision, may increase opportunities to screen for HIV-1 risk and initiate conversations about prevention options.^35^ In addition, although risk scores may prompt re-evaluation of self-perceived risk,^15,16^ self-perceived risk does not necessarily translate to PrEP uptake.^14^ Overall efforts to increase uptake for PrEP as part of an expansion of prevention options for women should also focus on barriers such as cost, accessibility, and stigma.^36^ Furthermore, both HIV-1 risk perception and empiric risk vary over time; future research should evaluate how risk scoring tools may support PrEP continuation decisions.

Our validation of the VOICE risk score showed similar predictive performance in this cohort as in other cohorts of women of similar age enrolled in clinical trials in sub-Saharan Africa.^9,19^ Although age-specific risk scores had moderate predictive performance, they performed only slightly better than the VOICE risk score applied to the full cohort of women aged 18–35, suggesting that, despite differences in risk factors across age groups, the added value of these age-specific risk scores to the existing VOICE risk score may be limited. Furthermore, data contributing to the development of both these risk scores and the VOICE risk score were collected in the context of clinical trials and so may be limited by the necessarily small amount of behavioral and clinical history data collected in such studies. Recent research in the United States demonstrated the added benefit of leveraging machine learning methods and the large amount of data available from electronic health records (EHR) by developing an HIV-1 risk score that outperformed simpler risk scores used to date.^37^ The SEARCH study applied machine learning approaches to a limited number of demographic variables, with modest improvements over model-based risk scores developed from the same set of candidate predictors (an AUC of 0.73 vs. 0.70), suggesting that richer data, such as that from EHR, are needed to improve risk score predictive performance.^38^ As use of EHR grows in the future in sub-Saharan Africa,^39^ their extensive data may inform development of more precise risk scores and potentially leverage nonsensitive health information to minimize discomfort in patient–provider interactions in discussing sensitive sexual behaviors. Automated approaches for calculating risk scores may also limit confusion by hiding counter-intuitive risk factors from the risk score calculation. For example, among women aged 18–24, use of condoms was positively associated with HIV-1 acquisition. Although this association may be explained by condom use as an indicator of self-perceived risk or less stable partnerships, its inclusion in the risk score may be confusing to providers given that it runs counter to standard HIV-1 prevention advice. EHR data may also provide an opportunity to externally validate these simplified risk scores in non‐clinical trial settings, a critical next step to understanding their practical utility.^6,40^

In summary, we identified important differences in the composite factors that best predict HIV-1 seroconversion among young women aged 18–24 and women aged 25–35 and highlighted the role of contextual factors in combination with individual-level factors in predicting HIV-1 risk. Nonetheless, the developed risk scores showed only modest improvement over existing non–age-specific risk scoring tools, indicating that the performance of existing screening tools would not be sufficiently improved by age stratification to warrant the added complexity of implementing age-specific risk scores. Overall risk of HIV-1 acquisition was high even among those with a low risk score, supporting high coverage of combination HIV-1 prevention for all but the lowest risk women. Approaches for targeted PrEP provision to women in South Africa may require more extensive data than are currently available to improve prediction.

## Supplementary Material

SUPPLEMENTARY MATERIAL
