# Supplementary material for: Age-Specific Risk Scores Do Not Improve HIV-1 Prediction Among Women in South Africa
Source: J Acquir Immune Defic Syndr. 2020 Jul 23;85(2):156–64. doi: 10.1097/QAI.0000000000002436 (PMC7495976; doi:10.1097/QAI.0000000000002436)
Supplement: SUPPLEMENTARY MATERIAL [file qai-85-156-s001.docx]

**Table S1**. HIV-1 incidence by risk score points and threshold values among women aged 18-24 and 25-35 in risk scores including or excluding laboratory-based sexually transmitted infection variables.

| Risk score points | HIV-1 incidence per 100 person-years (95% CI) | | | |
| --- | --- | --- | --- | --- |
|  | Ages 18-24 | | Ages 25-35 | |
|  | Including lab-based variables | Excluding lab-based variables | Including lab-based variables | Excluding lab-based variables |
| 0 | 0.0 (0.0, 0.0) | 0.0 (0.0, 0.0) | 0.0 (0.0, 0.0) | 0.0 (0.0, 0.0) |
| 1 | 1.3 (0.4, 3.5) | 2.0 (0.9, 4.1) | 0.0 (0.0, 0.0) | 0.0 (0.0, 0.0) |
| 2 | 2.5 (1.4, 4.5) | 2.6 (1.5, 4.2) | 1.1 (0.3, 3.9) | 1.9 (0.5, 6.9) |
| 3 | 3.6 (2.5, 5.1) | 5.6 (4.3, 7.2) | 0.5 (0.1, 2) | 1.6 (0.8, 3.0) |
| 4 | 4.9 (3.8, 6.3) | 5.4 (4s.3, 6.7) | 2.3 (1.3, 3.7) | 3.3 (2.3, 4.5) |
| 5 | 7.2 (5.4, 9.5) | 8.3 (5.6, 12.1) | 2.9 (1.9, 4.1) | 6.0 (4.3, 8.3) |
| 6 | 9.2 (5.8, 13.9) | 12.1 (7.4, 18.9) | 8.5 (5.9, 11.9) | -- |
| 7 | 12.5 (7.3, 20.1) | 10.7 (3.9, 25.8) | 12.0 (3.7, 33.6) | -- |
| 8 | 12.4 (6.4, 22.3) | -- | -- | -- |
| 9 | 15.5 (5.6, 37.2) | -- | -- | -- |
| 10 | 28.6 (8.8, 79.6) | -- | -- | -- |
| 11 | 0.0 (0.0, 0.0) | -- | -- | -- |
| < optimal threshold | 3.9 (3.2, 4.7) | 2.3 (1.4, 3.5) | 2.3 (1.7, 3.0) | 2.5 (1.8, 3.3) |
| ≥ optimal threshold | 8.8 (7.1, 10.7) | 6.1 (5.3, 7.1) | 8.6 (6.1, 12.0) | 6.0 (4.3, 8.3) |
| < alternative threshold | 2.9 (2.1, 4.0) | -- | -- | -- |
| ≥ alternative threshold | 6.7 (5.7, 7.9) | -- | -- | -- |

**Figure S1**. Receiver operating characteristic curves for the HIV-1 predictive performance of full risk scores and individual predictors among women aged 18-24 and 25-35.

**Figure S2**. Calibration of HIV-1 risk scores among women aged 18-24 and 25-35, as shown by observed and risk score-predicted cumulative HIV-1 incidence.

**Figure S3**. Cumulative HIV-1 incidence among women meeting the optimal discriminatory threshold value by age-specific risk score.

**Figure S4**. Comparison of receiver operating characteristic curves (ROC) for continuously-parameterized full multivariable risk models and risk scores by age and inclusion of laboratory variables. Similar ROC shapes and area under the ROC indicate that there is negligible loss of predictive performance when using simplified risk scores with rounded point values and categorical response options, compared to the full multivariable risk model.
